# The Neutrophil to Lymphocyte Ratio Is Associated With the Risk of Subsequent Dementia in the Framingham Heart Study

**DOI:** 10.3389/fnagi.2021.773984

**Published:** 2021-11-30

**Authors:** Jaime Ramos-Cejudo, Andrew D. Johnson, Alexa Beiser, Sudha Seshadri, Joel Salinas, Jeffrey S. Berger, Nathanael R. Fillmore, Nhan Do, Chunlei Zheng, Zanetta Kovbasyuk, Babak A. Ardekani, Omonigho M. Bubu, Ankit Parekh, Antonio Convit, Rebecca A. Betensky, Thomas M. Wisniewski, Ricardo S. Osorio

**Affiliations:** ^1^Department of Psychiatry, New York University Grossman School of Medicine, New York, NY, United States; ^2^VA Boston Cooperative Studies Program, MAVERIC, VA Boston Healthcare System, Boston, MA, United States; ^3^Population Sciences Branch, Division of Intramural Research, National Heart, Lung, and Blood Institute, Framingham, MA, United States; ^4^The Framingham Heart Study, Boston, MA, United States; ^5^Department of Biostatistics, Boston University School of Public Health, Boston, MA, United States; ^6^Department of Neurology, Boston University School of Medicine, Boston, MA, United States; ^7^Glenn Biggs Institute for Alzheimer’s and Neurodegenerative Diseases, University of Texas Health Science Center at San Antonio, San Antonio, TX, United States; ^8^Department of Neurology, Center for Cognitive Neurology, NYU Grossman School of Medicine, New York, NY, United States; ^9^Division of Vascular Surgery, Department of Surgery, NYU Grossman School of Medicine, New York, NY, United States; ^10^Division of Cardiology and Hematology, Department of Medicine, NYU Grossman School of Medicine, New York, NY, United States; ^11^Center for the Prevention of Cardiovascular Disease, NYU Grossman School of Medicine, New York, NY, United States; ^12^Harvard Medical School, Boston, MA, United States; ^13^Boston University School of Medicine, Boston, MA, United States; ^14^Nathan Kline Institute, Orangeburg, NY, United States; ^15^Division of Pulmonary, Critical Care and Sleep Medicine, Icahn School of Medicine at Mount Sinai, New York, NY, United States; ^16^Department of Biostatistics, New York University School of Global Public Health, New York, NY, United States; ^17^Department of Pathology, NYU Grossman School of Medicine, New York, NY, United States

**Keywords:** Alzheimer’s disease, dementia, Framingham, FHS, neutrophil to lymphocyte ratio, NLR, complete blood count (CBC), risk prediction

## Abstract

**Objective:** Active neutrophils are important contributors to Alzheimer’s disease (AD) pathology through the formation of capillary stalls that compromise cerebral blood flow (CBF) and through aberrant neutrophil signaling that advances disease progression. The neutrophil to lymphocyte ratio (NLR) is a proxy of neutrophil-mediated inflammation, and higher NLR is found in persons diagnosed with clinical AD. The objective of this study was to investigate whether increased NLR in older adults is independently associated with the risk of subsequent dementia.

**Methods:** We examined associations of baseline NLR with incident dementia risk in the community-based Framingham Heart Study (FHS) longitudinal cohorts. The association between NLR and risk of dementia was evaluated using the cumulative incidence function (CIF) and inverse probability-weighted Cox proportional cause-specific hazards regression models, with adjustment for age, sex, body mass index (BMI), systolic and diastolic blood pressure, diabetes, current smoking status, low-density lipoprotein (LDH), high-density lipoprotein (LDL), total cholesterol, triglycerides, and history of cardiovascular disease (CVD). Random forest survival models were used to evaluate the relative predictive value of the model covariates on dementia risk.

**Results:** The final study sample included 1,648 participants with FHS (average age, 69 years; 56% women). During follow-up (median, 5.9 years), we observed 51 cases of incident dementia, of which 41 were AD cases. Results from weighted models suggested that the NLR was independently associated with incident dementia, and it was preceded in predictive value only by age, history of CVD, and blood pressure at baseline.

**Conclusion:** Our study shows that individuals with higher NLR are at a greater risk of subsequent dementia during a 5.9-year follow-up period. Further evaluating the role of neutrophil-mediated inflammation in AD progression may be warranted.

## Introduction

The variability of amyloid-beta (Aβ) and tau pathology associated with clinical symptoms reflects that additional factors influence the progression of cognitive decline in Alzheimer’s disease (AD). Vascular dysfunction and inflammation have been implicated in the pathogenesis of AD in the earliest stages of the disease prior to significant Aβ and tau accumulation (Iturria-Medina et al., 2016; Love and Miners, 2016; Nortley et al., 2019). In addition, clinical studies have indicated that the inflammatory status influences the rate of disease progression in patients with AD (Holmes et al., 2009; Cunningham and Hennessy, 2015).

Recently, neutrophils have been identified as potential key elements of innate immunity contribution to the disease, and a hyperactive neutrophil state has been found in patients with AD and has been associated with clinical progression (Dong et al., 2018). Neutrophils are the most abundant leukocyte type in the human blood, and although the best characterized function of neutrophils in the defense against infectious pathogens, neutrophils are implicated in the repair of both infectious and sterile injuries (Kruger et al., 2015). Results in animal models of AD have suggested that neutrophils may be implicated in the breakdown of the blood-brain barrier (BBB) and recruited in the brain parenchyma through the integrin LFA-1 predominantly in perivascular regions with Aβ deposition (Baik et al., 2014; Zenaro et al., 2015). A potential role of neutrophils in the hyperphosphorylation of tau has also been suggested (Zenaro et al., 2015; Nemeth et al., 2020).

Neutrophils are known to have a very short life cycle ranging from just a few hours to up to 5–6 days in circulation (Pillay et al., 2010). During normal inflammation, neutrophils are cleared from the circulation once the initial inflammatory insult is resolved. In aging, however, inaccurate neutrophil chemotaxis may lead to compromised clearance of neutrophils, potentially contributing to a chronic inflammation state with reduced formation of neutrophil extracellular traps (NET), phagocytic response, and reactive oxygen species (ROS) production (Tseng and Liu, 2014). These abnormalities may partially explain the contribution of neutrophil adhesion to brain capillaries in the formation of capillary stalls and reduction in cerebral blood flow (CBF) that is observed in animal models (Cruz Hernandez et al., 2019; El Amki et al., 2020), a mechanism that could occur at the early stages in aging and influence the pathological accumulation of Aβ and tau when sustained over years.

One of the most widely studied and available clinical markers of peripheral inflammation is the neutrophil to lymphocyte ratio (NLR), which has been associated with poor prognosis in several cancer outcomes (Walsh et al., 2005; Sarraf et al., 2009), diabetes (Imtiaz et al., 2012), and cardiovascular disease (CVD) (Bhat et al., 2013). Almost a decade ago, a higher NLR was first reported in patients with AD (Kuyumcu et al., 2012) and replicated in a study using longitudinal data and repetitive measures over time (Rembach et al., 2014). The higher NLR levels observed in patients with AD were associated with increased amyloid burden, but this association was no longer present after adjustment for age, sex, and ApoE4 status, thus potentially limiting the potential of NLR as a diagnostic biomarker in more advanced stages of the disease. Because of the hyperactive neutrophil state identified in patients with AD and the higher NLR ratios observed in AD versus controls, it is possible that higher NLR ratios in elderly adults may be independently associated with the risk of incident dementia, although, such an association has yet to be identified.

The aim of this study was to examine whether baseline NLR was independently associated with incident dementia in the Framingham Heart Study (FHS), a longitudinal, community-based cohort with rigorous and continuous surveillance for clinical dementia incidence and a large number of participants with NLR measures.

## Materials and Methods

### Standard Protocol Approvals, Registrations, and Patient Consents

All participants provided written informed consent. Study protocols and consent forms were approved by the institutional review board at the Boston University Medical Center.

### The Framingham Heart Study

The FHS is one of the few historical active longitudinal cohort studies in the United States, initiated in 1948 and with over 70 years of follow-up evaluations. Following recruitment, the original cohort of 5,209 residents from Framingham, MA, underwent up to 32 examinations every 2 years, where various clinical and laboratory data were collected (Dawber et al., 1963). In 1971, a total of 5,124 children of the original cohort and their spouses were enrolled in the Offspring cohort who completed a total of nine examinations, with the latest examination performed in 2011–2014 (Kannel et al., 1979). *Offspring* cohort participants who attended the ninth examination cycle (2011–2014), during which a complete blood count (CBC) was obtained, were eligible for the present investigation.

### Blood Collection and Complete Blood Count

Participants were asked to fast after 8:00 p.m., the evening before their clinic exam, and considered to be fasting after a minimum of a 10-h fast. Blood was drawn from participants in a supine position, using standard venipuncture technique, typically between 7:00 a.m. and 9:00 a.m. Hematology testing was performed using whole blood [Tyco Monoject, 15% ethylenediaminetetraacetic acid, EDTA, (K3)]. A CBC with differential was performed on EDTA whole blood using a Beckman Coulter HmX Hematology Analyzer as described previously (Sloan et al., 2015). Blood collection was performed following the exact same procedures in every subject. Hematology testing included estimation of intra-assay CV in ∼10% samples run in duplicate. The NLR was defined as the absolute neutrophil count by the absolute lymphocyte count from the CBC panel. Assessment of cognitive decline and dementia was performed by a panel blinded to the CBC/NLR results.

### Dementia Characterization in the Framingham Heart Study

The main outcome of the present study was an incident clinical diagnosis of dementia using continuous surveillance until the conclusion of the follow-up period up to 2018. The diagnosis of dementia was based on criteria from the *Diagnostic and Statistical Manual of Mental Disorders*, fourth edition (DSM-IV). The diagnosis of AD was based on criteria for possible, probable, or definite AD from the National Institute of Neurological and Communicative Disorders and Stroke and the AD and Related Disorders Association (NINCDS–ADRDA). Additional methods for continuous surveillance of dementia and AD in the FHS have been previously described (Seshadri et al., 1997, 2011; Au et al., 2012; Satizabal et al., 2016). In brief, the cognitive function of all participants was assessed using the Mini-Mental State Examination (MMSE) (Folstein et al., 1975) at every examination. The MMSE was used to flag participants for a dementia panel review based on their performance if any of the following occurred: (a) an absolute score <23 for all persons, (b) score <24 for persons with only high school completed, (c) score <26 for participants with a college education, (d) >3-point decline between successive examinations, or (e) >5-point decline from the highest obtained MMSE scores of the participants. Participants were also flagged for additional evaluation based on concerns from participants themselves, relatives, or other professionals. In additional visits, flagged participants had a full neuropsychological and neurological examination, which was also reviewed to refer for dementia evaluation by the dementia review panel. The panel, which includes neurologists (JS and SS) and neuropsychologists, used data from multiple sources to assess possible cognitive decline and dementia to determine whether a participant had dementia, the dementia subtype, and the date of diagnosis. After a participant died, a medical panel manually reviewed medical records up to the date of death to assess for the potential cognitive decline since the last examination of the participants. This medical panel referred any participants who may have presented with a cognitive decline for postmortem review by the dementia review panel [refer to Satizabal et al. (2016) – Supplementary Material, for a detailed description of dementia surveillance in the FHS]. The main outcome of the present study was incident dementia using continuous surveillance with clinician diagnosis at the conclusion of the follow-up period up to 2018.

### Demographic and Clinical Covariates

Clinic examination corresponding to the CBC (examination cycle 9) was defined as the baseline. Smoking was defined based on smoking status prior to the year of baseline. We defined diabetes mellitus by fasting glucose levels above 126 mg/dl (7.0 mmol/L) and/or use of antidiabetic treatments. Levels of all cardiovascular risk variables, including body mass index (BMI), total cholesterol, high-density lipoprotein (HDL) cholesterol, and triglycerides, were determined from examination cycle 9. History of CVD events at the time of clinical examination included: reported history of coronary heart disease (CHD), congestive heart failure (CHF), myocardial infarct (MI), intermittent claudication (IC), ischemic stroke, intracerebral hemorrhage, or transient ischemic attack (TIA).

### Statistical Analysis

R 3.6 and Python 3.7 were used for statistical analysis and visualization. The analysis involved the following variables: (a) the NLR; (b) outcome: clinical dementia diagnosis; and, (c) basic demographic and clinical parameters (continuous or categorical), which included age, sex, BMI, smoking, HDL, low-density lipoprotein (LDL), total cholesterol, triglycerides, systolic and diastolic blood pressure, diabetes, and history of CVD. Univariate analysis of demographic and clinical differences between study groups included *t*-tests for continuous variables and χ^2^ tests for nominal variables. Kruskal–Wallis tests were applied to non-normal distributions. Spearman correlations were used to test the association between continuous variables. Generally, we defined statistical significance by *p* < 0.05, and tendencies by *p* ≤ 0.1.

Follow-up duration was calculated from the date of the CBC until the most recently available clinical diagnosis prior to the end of follow-up in 2018. To detect potentially independent associations between the NLR and incident dementia, we fit Cox proportional hazard models with inverse probability weighting (IPW). The IPW method weighted each subject by the inverse of the probability of their observed NLR using the median as a cutoff, adjusting for non-random selection of participants into high versus low NLR groups. A logistic regression model for high versus low NLR, adjusted for age, sex, BMI, diabetes, current smoking status, LDH, LDL, total cholesterol, triglycerides, systolic and diastolic blood pressure, and history of CVD was used to estimate these probabilities. The Cox models were then weighted by the inverse of the probabilities of the NLR, adjusted for the above covariates. The proportional hazards assumption was validated using the Schoenfeld residual test included in the cox.zph function of the coxph package (Grambsch, 1995; In and Lee, 2019). Next, using the propensity score model for low versus high NLR (split at the median) for all participants and the causalCmprsk package (Vakulenko-Lagun et al., 2020), we estimated the cumulative incidence function (CIF) for dementia for the two groups. Due to the competing risk of death, the models can be interpreted as cause-specific hazard models (i.e., for the risk of dementia among those still alive). Finally, in an exploratory approach, we applied the survival data implementation of Breiman’s random forest models in the randomForestSRC (O’Brien et al., 2021) to estimate the relative importance of each of the covariates in dementia risk. Briefly, the variable importance of each predictor is estimated by using variable selection methods of random forest survival models. The variable selection method uses a prediction error approach by “noising-up” each variable in turn. The variable importance of a variable X_i_ is the difference in prediction error when X_i_ is randomly permuted, compared to the prediction error under the true values. The package ggRandomForests was used for visualization. Code is available upon reasonable request and for collaboration and reproducibility purposes. The data are available in the BioLINCC repositories.

## Results

### Study Sample

**FIGURE 1:**
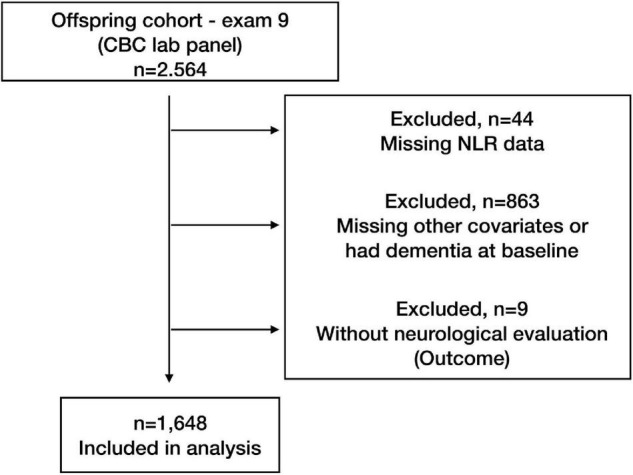
Inclusion diagram.

The final study sample consisted of 1,648 FHS participants and included the combination of NLR and clinical and demographic covariates (Figure 1) described previously. Table 1 summarizes baseline characteristics for all participants at the time of the CBC. The average age of participants in the study at baseline was 69 years [the interquartile range (IQR): 64, 76]; 55.8% were female (920).

**TABLE 1:** Comparison of demographic and clinical characteristics by the neutrophil to lymphocyte ratio (NLR).

	Overall (*n* = 1,648)	NLR below median (*n* = 826)	NLR above median (*n* = 822)	*P*-value
Age, median [Q1, Q3]	69.0 [64.0, 76.0]	68.0 [63.0, 74.0]	71.0 [65.0, 77.0]	<0.001
Female sex, *n* (%)	920 (55.8)	515 (62.3)	405 (49.3)	<0.001
BMI, median [Q1, Q3]	27.7 [24.7, 30.9]	27.5 [24.3, 30.5]	28.0 [25.1, 31.4]	0.006
LDL cholesterol, median [Q1, Q3]	96.0 [78.0, 118.0]	101.5 [81.0, 122.0]	93.0 [75.0, 112.0]	<0.001
HDL cholesterol, median [Q1, Q3]	60.0 [49.0, 74.0]	61.0 [50.0, 76.0]	58.0 [47.0, 71.0]	<0.001
Total cholesterol, median [Q1, Q3]	183.0 [158.8, 207.0]	188.0 [163.2, 211.0]	177.0 [153.0, 202.0]	<0.001
Triglycerides, median [Q1, Q3]	99.0 [74.0, 133.0]	96.0 [72.0, 132.0]	100.5 [76.0, 134.0]	0.041
Smoker, *n* (%)	78 (4.7)	32 (3.9)	46 (5.6)	0.126
Diabetes, *n* (%)	159 (9.6)	76 (9.2)	83 (10.1)	0.594
Systolic BP, median [Q1, Q3]	125.0 [115.0, 137.0]	125.0 [113.0, 136.0]	126.0 [116.0, 137.0]	0.011
Diastolic BP, median [Q1, Q3]	72.0 [65.0, 78.0]	72.0 [66.0, 78.0]	71.0 [65.0, 78.0]	0.176
CVD History, *n* (%)	295 (17.9)	122 (14.8)	173 (21.0)	0.001
NLR, median [Q1, Q3]	2.3 [1.8, 3.0]	1.8 [1.4, 2.0]	3.0 [2.6, 3.8]	<0.001
Number of dementia cases, *n* (%)	51 (3.1)	16 (1.9)	35 (4.3)	0.01
Number of deaths, *n* (%)	92 (5.6)	33 (4.0)	59 (7.2)	0.007

*BMI, body mass index; BP, blood pressure; LDL, low-density lipoprotein; HDL, high-density lipoprotein; Chol, cholesterol; CVD history, history of cardiovascular events. Definitions described in section “Materials and Methods.”*

### Neutrophil to Lymphocyte Ratio

Intra-assay CVs were <2.5% for both the total neutrophil and lymphocyte counts. The absolute neutrophil and lymphocyte count and the NLR followed a unimodal distribution.

Although the participants with a diagnosis of dementia at baseline were excluded from the analysis, a brief comparison of the clinical labs at baseline indicated higher neutrophil counts and NLR in dementia cases (Supplementary Figure 1). In the final cohort (non-dementia) participants that showed a higher NLR (above median) were older and predominantly male; they had lower LDL, HDL, and total cholesterol and higher BMI, triglycerides, systolic blood pressure, and rates of CVD history at baseline (*p* < 0.05; Table 1).

### Association of the Neutrophil to Lymphocyte Ratio and Incident Dementia

**FIGURE 2:**
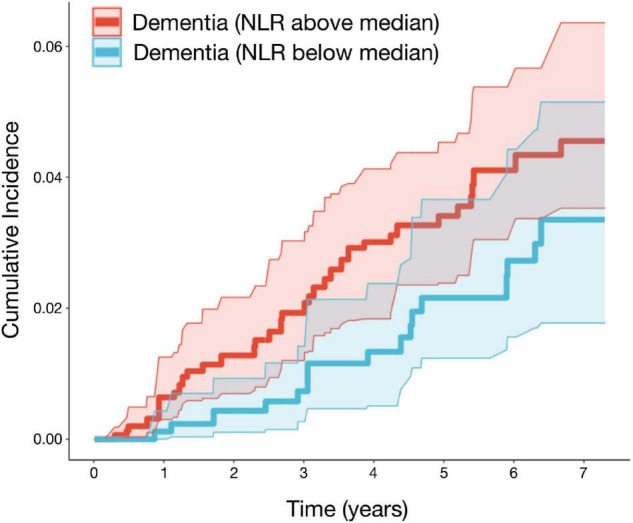
The neutrophil to lymphocyte is associated with higher rates of incident dementia in the FHS. Adjusted cumulative incidence functions and 95% CIs for dementia for the NLR groups (defined as above/below median). Higher NLR at baseline was associated with a greater incidence of dementia. Models were adjusted for age, sex, BMI, systolic and diastolic blood pressure, diabetes, current smoking status, HDL, LDL, total cholesterol, triglycerides, and history of CVD (*n* = 1,647; events = 51; median follow-up = 5.9 years). BMI, body mass index; LDL, low-density lipoprotein; HDL, high-density lipoprotein; CVD, cardiovascular disease; FHS, Framingham Heart Study.

**FIGURE 3:**
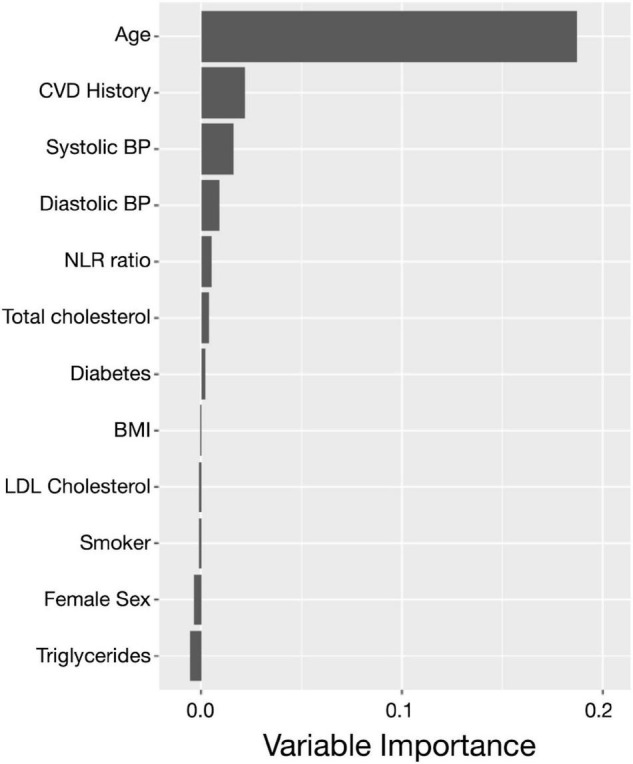
The neutrophil to lymphocyte ratio plays an important role in incident dementia prediction. Ranked feature importance for random forest survival models for the prediction of incident dementia assigned a higher priority for the NLR (coded as continuous) over other clinical covariates. The highest priority was given to age, history of CVD, and hypertension, followed by the NLR and the remaining clinical and demographic covariates. Variable importance close to zero indicates the variable contributed nothing to predictive accuracy, and negative values indicate the predictive accuracy improved when the variable was misspecified and therefore was not informative. CVD, cardiovascular disease; FHS, Framingham Heart Study; NLR, neutrophil to lymphocyte ratio.

Of the 1,648 participants, 51 cases of incident dementia (41 confirmed AD cases) and 85 deaths without incident dementia were observed during a median follow-up of 5.9 years (IQR: 2.6, 6.9). Logistic models used to estimate IPW weights indicated strong associations between the NLR and age, male sex, BMI, and smoking status (Supplementary Table 1). Although the CIs are overlapping, and there is insufficient power to conclude that the incidence curves are different, CIF curves suggested that groups with a higher baseline NLR demonstrated a greater incidence of dementia (Figure 2). For example, the probability of dementia prior to death occurring within 5.9 years for those with low NLR at baseline is 2.73% [95% CI: 1.56, 4.43], while for those with high NLR it was 4.11% [95% CI: 3.05, 5.38]. Ranked feature importance of random forest competing for risk models assigned highest priority to age, systolic and diastolic blood pressure, and history of CVD, immediately followed by the NLR (Figure 3). Variables with lower importance than the NLR included total cholesterol, diabetes, BMI, LDL cholesterol, smoking status, sex, and triglycerides. Adjusted hazard ratios (HRs) for the IPW multivariate Cox proportional hazard models for incident dementia are summarized in Supplementary Tables 2, 3. Higher NLR at baseline was independently associated with incident dementia when included as a continuous variable (HR: 1.22; 95% CI: 1.05, 1.43; *p* = 0.01). Results indicated a 22% increase in dementia risk per unit increase in the NLR. Elevated HRs are also apparent with respect to categorical coding of NLR: above versus below the median (HR: 1.80; 95% CI: 0.96, 3.37; *p* = 0.07) in adjusted models. A sensibility analysis inspecting confirmed AD cases as an outcome similarly suggested elevated NLR was associated with higher rates of incident AD (Supplementary Tables 4, 5 and Supplementary Figure 2).

## Discussion

Recently, neutrophils have been identified as potential innate immunity players contributing to AD pathology, and a hyperactive neutrophil state has been found in patients with AD and associated with AD progression. The NLR serves as a composite inflammatory biomarker, which incorporates information from two leukocyte subtypes. Exploration of the NLR as a proxy for neutrophil-mediated inflammation is of interest since the NLR can be obtained as part of the standard CBC panel, which is widely available in most hospitals. Our study in the community-based longitudinal FHS Offspring cohort demonstrated that individuals with elevated NLR are at a greater risk of dementia during a 5.9-year follow-up period. These findings persist after adjustment for a number of covariates that could play a confounding role in the association of NLR with dementia risk. Importantly, results from the sensitivity analysis suggested an association of the NLR and AD risk specifically.

The role of neutrophils in AD pathology and clinical progression has been previously suggested (Shad et al., 2013; Pietronigro et al., 2017; Dong et al., 2018; Stock et al., 2018; Bawa et al., 2020; Rossi et al., 2021). Although variations in the NLR have been associated with several clinical outcomes other than AD (Walsh et al., 2005; Sarraf et al., 2009; Imtiaz et al., 2012; Bhat et al., 2013), the interpretation of a change in the NLR remains controversial. Studies in animal models suggest that clearance of neutrophils may be reduced with aging, potentially compromising inflammation resolution following injury. The increased NLR with aging in humans may be indicative of such a process and whether abnormal neutrophil phenotypes are associated with the increased NLR should be studied in the future. Recent studies show that neutrophils may be involved in capillary stall formation and contribute to CBF reductions that when sustained over time may lead to oxidative stress, endothelial damage and influence the pathological accumulation of Aβ and tau at the early stages of the disease (Cruz Hernandez et al., 2019; El Amki et al., 2020). Additionally, the vascular dysfunction state that is associated with AD may increase endothelial signaling facilitating neutrophil recruitment to the capillary vessel walls in a feed-forward loop. Studies need to evaluate whether the stall formations are associated with increased NLR.

Although our primary goal was to evaluate the association of the NLR with incident dementia, we also observed higher NLR in the dementia cases at baseline that was excluded from analysis when compared to dementia-free participants. The results are in agreement with previous research that identified higher NLR in patients with AD when compared to age-matched controls (Kuyumcu et al., 2012; Rembach et al., 2014). The NLR ratio has also been associated with conditions related to AD risk and influencing progressions, such as CVD (Angkananard et al., 2018; Kim et al., 2018; Haybar et al., 2019), obstructive sleep apnea (Altintas et al., 2015; Sunbul et al., 2015; Oyama et al., 2016; Rha et al., 2020), depression (Aydin Sunbul et al., 2016; Demircan et al., 2016; Arabska et al., 2018; Liang et al., 2019), and obesity (Rodriguez-Rodriguez et al., 2020). Our findings are therefore consistent with previous research that identified an association of the NLR with AD and related risk factors.

In observational studies like the FHS where there is no random assignment to treatment groups (or variable of interest like the NLR in this case), the unadjusted comparison between treatment groups may be misleading due to confounding. To adjust for measured confounders in our study, we used inverse probability of treatment weighting which is a robust approach for correcting for potential confounding (Neumann and Billionnet, 2016; Vakulenko-Lagun et al., 2020). In our study using weighted Cox models in elderly adults and a 5.9-year median follow-up suggested that the NLR was independently associated with incident dementia. Additionally, the variable importance results of random forest survival models suggested that the NLR had a considerable high predictive value when compared to other variables known to be relevant in dementia risks, such as age, sex, BMI, blood pressure, diabetes, smoking status, lipids, and history of CVD, suggesting that the NLR may add predictive value when incorporated in machine-learning models for dementia and this needs to be examined in future studies.

Our study has several limitations. First, although we adjusted for a relatively large number of covariates, we did not exclude/adjust for psychiatric and autoimmune disorders known to increase dementia risk that also to may influence the NLR ratios (e.g., depression) and future analysis should control for these conditions and look at each of the risk groups separately. Additionally, the number of dementia cases available for risk stratification in our study was relatively small and future studies should replicate our analysis if a larger number of incipient dementia cases are available. Second, in our primary analysis, we did not control for the number of years of education, the use of hypertensive drugs, and drugs potentially modulating the CBC results due to a considerable number of missing observations that limited the number of incipient dementia cases available for analysis. A sensitivity analysis also including the aforementioned variables showed associations with the same directionality of NLR associated with increased risk of incipient dementia. Additionally, our study did not account for the ApoE4 status, which is the main genetic risk factor for AD (Tsai et al., 1994; Kim et al., 2009; Liu et al., 2013) and previous research found that the ApoE4 status influences the NLR (Rembach et al., 2014). Cholesterol homeostasis has a pivotal function in regulating immune cells, and the role of ApoE in neutrophil-mediated inflammation has been suggested in experimental models (Terkeltaub et al., 1991; Barger and Harmon, 1997; Zhou et al., 2019; Doring et al., 2020).

## Conclusion

Our study in 1,648 participants of a well-characterized community-based cohort shows an independent association of the NLR with future dementia, reinforcing the role of neutrophil-mediated inflammation in AD pathology and progression.

## Future Perspectives

Future studies need to determine whether the increased NLR observed in certain participants at higher risk of dementia is independent or mediated by the ApoE4 status and the role of other AD-risk factors in the association. Additionally, dementia is more frequent in women and some race and ethnic groups, and we were not powered to perform disaggregated analyses. Finally, whether the NLR is associated with other markers of vulnerability to cognitive decline (global cognition, MRI, PET, and AD-biofluid markers) in middle age needs to be further investigated.

## Data Availability Statement

Publicly available datasets were analyzed in this study. This data can be found here: https://biolincc.nhlbi.nih.gov/home/.

## Ethics Statement

The studies involving human participants were reviewed and approved by the Boston University Medical Center. The patients/participants provided their written informed consent to participate in this study.

## Author Contributions

JR-C designed and conceptualized the study, analyzed the data, performed the statistical analysis, and drafted the manuscript. AJ, AB, SS, and JS interpreted the data, major role in the acquisition of data, and revised the manuscript for intellectual content. JB, NF, ND, CZ, ZK, OB, AP, AC, RB, and TW revised the manuscript for intellectual content. TW and RO designed and conceptualized study, interpreted the results, and revised the manuscript for intellectual content. All authors contributed to the article and approved the submitted version.

## Author Disclaimer

The views expressed in this manuscript are those of the authors and do not necessarily represent the views of the National Heart, Lung, and Blood Institute, National Institutes of Health, or United States Department of Health and Human Services.

## Conflict of Interest

The authors declare that the research was conducted in the absence of any commercial or financial relationships that could be construed as a potential conflict of interest.

## Publisher’s Note

All claims expressed in this article are solely those of the authors and do not necessarily represent those of their affiliated organizations, or those of the publisher, the editors and the reviewers. Any product that may be evaluated in this article, or claim that may be made by its manufacturer, is not guaranteed or endorsed by the publisher.

## Abbreviations

AD: Alzheimer’s disease
NLR: neutrophil to lymphocyte ratio
CI: confidence interval
CVD: cardiovascular disease
DSM-IV, Diagnostic and Statistical Manual of Mental Disorders: 4th edition
FHS: Framingham Heart Study
HR: hazard ratio
MMSE: Mini-Mental State Examination
CBC: complete blood count.
