# Supplementary material for: The Neutrophil to Lymphocyte Ratio Is Associated With the Risk of Subsequent Dementia in the Framingham Heart Study
Source: Front Aging Neurosci. 2021 Nov 30;13:773984. doi: 10.3389/fnagi.2021.773984 (PMC8670436; doi:10.3389/fnagi.2021.773984)
Supplement: Supplementary file 1 [file Data_Sheet_1.docx]

**SUPPLEMENTARY TABLES**

**Supplementary Table 1** – Results of logistic model for propensity score weight estimation.

|  | **Estimate** | **Std. Error** | **P-Value** |
| --- | --- | --- | --- |
| Intercept | -2.999192 | 0.844734 | 0.000385 *** |
| **Age** | **0.037093** | **0.007532** | **8.46e-07 ***** |
| **Female Sex** | **-0.472107** | **0.117927** | **6.24e-05 ***** |
| **BMI** | **0.026286** | **0.010499** | **0.012291 *** |
| Systolic BP | 0.004766 | 0.003913 | 0.223172 |
| Diastolic BP | -0.003605 | 0.006999 | 0.606536 |
| **Smoker** | **0.640607** | **0.244478** | **0.008785 **** |
| LDL Cholesterol | 0.034582 | 0.178212 | 0.846138 |
| HDL Cholesterol | 0.039074 | 0.178226 | 0.826464 |
| Total Cholesterol | -0.039822 | 0.178176 | 0.823147 |
| Triglycerides | 0.009063 | 0.035649 | 0.799320 |
| Diabetes | -0.224744 | 0.179765 | 0.211222 |
| CVD History | 0.076110 | 0.141392 | 0.590375 |

**Supplementary Table 2 -** Results of inverse probability weighted (IPW) Cox proportional cause-specific hazards regression models for incident dementia, with adjustment for demographic and clinical covariates. The neutrophil to lymphocyte ratio (NLR) is coded as continuous.

| Outcome: Dementia | **Adjusted HR** | **95% CI** | **P-Value** |
| --- | --- | --- | --- |
| **Age** | **1.1680** | **1.1241, 1.2137** | **1.92e-15 ***** |
| Female Sex | 0.8539 | 0.4651, 1.5679 | 0.6105 |
| BMI | 0.9985 | 0.9411, 1.0595 | 0.9614 |
| Systolic BP | 0.9844 | 0.9644, 1.0047 | 0.1317 |
| Diastolic BP | 0.9941 | 0.9648, 1.0243 | 0.6977 |
| Smoker | 1.0831 | 0.2123, 5.5245 | 0.9235 |
| **LDL Cholesterol** | **0.3419** | **0.1257, 0.9297** | **0.0355 *** |
| **HDL Cholesterol** | **0.3395** | **0.1249, 0.9228** | **0.0342 *** |
| **Total Cholesterol** | **2.9419** | **1.0829, 7.9923** | **0.0343 *** |
| **Triglycerides** | **0.8065** | **0.6603, 0.9852** | **0.0352 *** |
| Diabetes | 0.4463 | 0.1255, 1.5868 | 0.2126 |
| **CVD History** | **1.8203** | **0.9183, 3.6083** | **0.0862 *** |
| **NLR** | **1.2219** | **1.0479, 1.4249** | **0.0106 *** |

**Supplementary Table 3** – Hazards ratios for the association of the neutrophil to lymphocyte ratio (NLR) to incident dementia.

|  | Adjusted HR | 95% CI | P-Value |
| --- | --- | --- | --- |
| Outcome: dementia; n =1,648, No. events = 51, median follow up=5.9-years | | | |
| NLR (continuous) | **1.22** | **1.05, 1.43** | **0.01** |
| NLR (above/below median) | **1.80** | **0.96, 3,37** | **0.07** |

Multivariate adjusted COX models with inverse probability weighting (IPW). All models adjusted for age, sex, BMI, blood pressure, diabetes, LDL, HDL, total cholesterol, triglycerides, current smoking status, and history of CVD events. Q = quartiles; T= tertiles. Associations with p<0.1 are reported in bold.

**Supplementary Table 4 -** Results of inverse probability weighted (IPW) Cox proportional cause-specific hazards regression models for incident AD, with adjustment for demographic and clinical covariates. The neutrophil to lymphocyte ratio (NLR) is coded as continuous.

| Outcome: AD | **Adjusted HR** | **95% CI** | **P-Value** |
| --- | --- | --- | --- |
| **Age** | **1.1746** | **1.12686, 1.2244** | **2.96e-14 ***** |
| Female Sex | 0.9879 | 0.48904, 1.9956 | 0.9729 |
| BMI | 0.9848 | 0.91844, 1.0560 | 0.6671 |
| Systolic BP | 0.9832 | 0.96182, 1.0050 | 0.1290 |
| Diastolic BP | 0.9963 | 0.96232, 1.0315 | 0.8340 |
| Smoker | 1.4210 | 0.26572, 7.5987 | 0.6813 |
| **LDL Cholesterol** | **0.2778** | **0.08920, 0.8650** | **0.0271 *** |
| **HDL Cholesterol** | **0.2774** | **0.08912, 0.8633** | **0.0269 *** |
| **Total Cholesterol** | **3.6076** | **1.15767, 11.2424** | **0.0269 *** |
| **Triglycerides** | **0.7735** | **0.61633, 0.9707** | **0.0267 *** |
| Diabetes | 0.3802 | 0.08228, 1.7573 | 0.2157 |
| **CVD History** | **1.9527** | **0.89841, 4.2443** | **0.0911 *** |
| **NLR** | **1.1974** | **0.97256, 1.4742** | **0.0896 *** |

**Supplementary Table 5** – Hazards ratios for the association of the neutrophil to lymphocyte ratio (NLR) to incident AD.

|  | Adjusted HR | 95% CI | P-Value |
| --- | --- | --- | --- |
| Outcome: AD; n =1,648, No. events = 41, median follow up=5.9-years | | | |
| **NLR (continuous)** | **1.20** | **0.97, 1.47** | **0.09** |
| NLR (above/below median) | 1.54 | 0.76, 3.10 | 0.23 |

Multivariate adjusted COX models with inverse probability weighting (IPW). All models adjusted for age, sex, BMI, blood pressure, diabetes, LDL, HDL, total cholesterol, triglycerides, current smoking status, and history of CVD events. Q = quartiles; T= tertiles. Associations with p<0.1 are reported in bold.

**Supplementary Figure S1** - **Histograms for the total neutrophil, lymphocyte counts, as well as the neutrophil to lymphocyte ratio (NLR) for all participants at baseline.** Participants with dementia at baseline (red) had higher neutrophil counts and NLR ratios when compared to non-dementia (green) (P<0.05). Participants with dementia at baseline were excluded from the survival analysis.

**
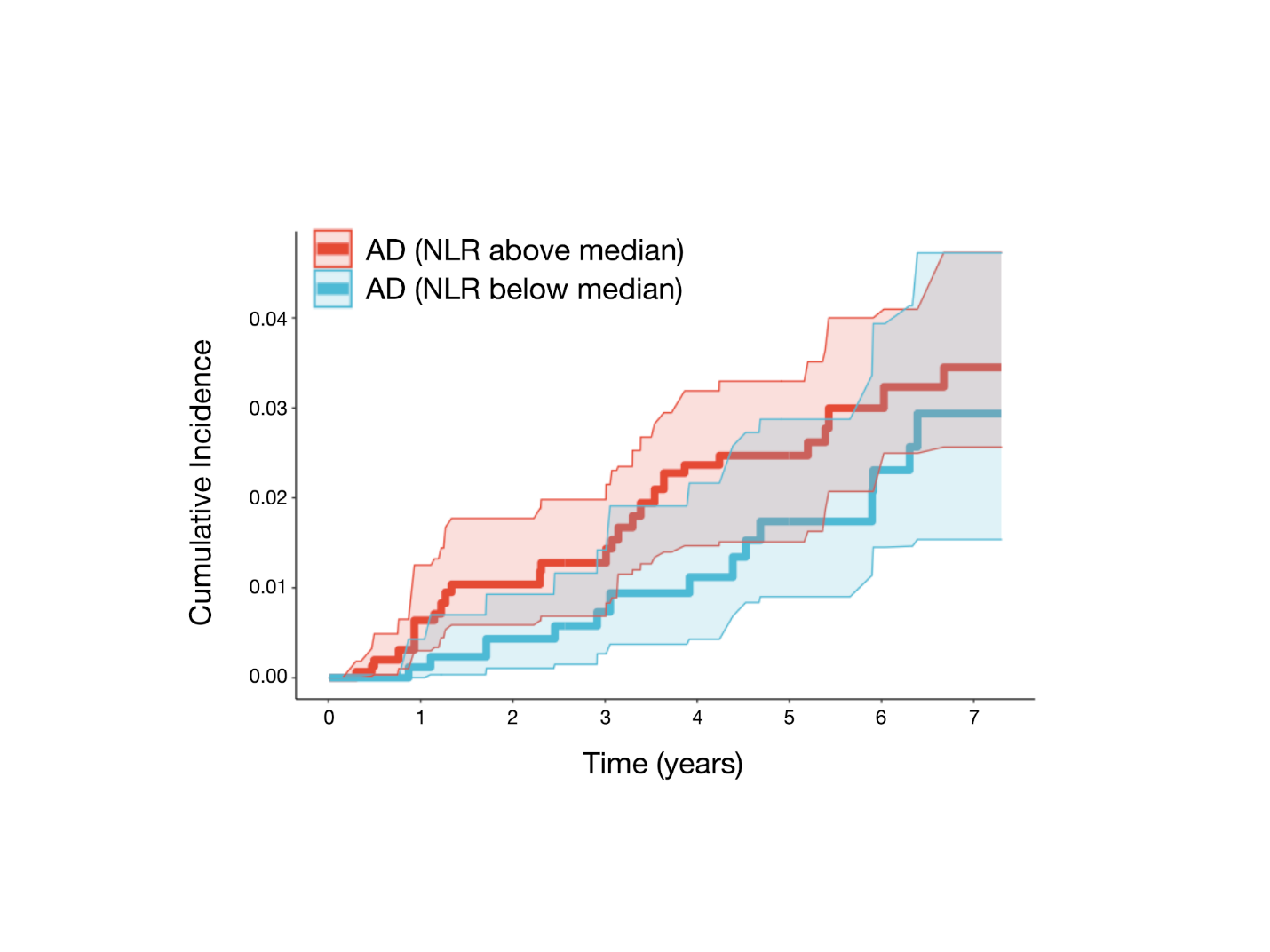
**

**Supplementary Figure S2** **–The neutrophil to lymphocyte is associated with higher rates of incident AD in the FHS.** Adjusted cumulative incidence functions and 95% confidence intervals for AD for the NLR groups (defined as above/below median). Higher NLR at baseline was associated to greater incidence of AD. Models were adjusted for age, sex, BMI, systolic and diastolic blood pressure, diabetes, current smoking status, HDL, LDL, total cholesterol, triglycerides, and history of CVD (n=1,647; Events=41; Median follow up = 5.9 years).
